# NADPH Oxidases in Diastolic Dysfunction and Heart Failure with Preserved Ejection Fraction

**DOI:** 10.3390/antiox11091822

**Published:** 2022-09-16

**Authors:** James P. Teuber, Kobina Essandoh, Scott L. Hummel, Nageswara R. Madamanchi, Matthew J. Brody

**Affiliations:** 1 Department of Pharmacology, University of Michigan, Ann Arbor, MI 48109, USA; 2 Department of Internal Medicine, University of Michigan, Ann Arbor, MI 48109, USA; 3 Ann Arbor Veterans Affairs Health System, Ann Arbor, MI 48105, USA

**Keywords:** HFpEF, heart failure, diastolic dysfunction, ROS, oxidative stress, redox signaling, NADPH oxidases, NOX2, NOX4, Rac1, nitrosative stress, S-nitrosylation, cardiomyopathy, cardiac hypertrophy, angiotensin, RAAS

## Abstract

Nicotinamide adenine dinucleotide phosphate (NADPH) oxidases regulate production of reactive oxygen species (ROS) that cause oxidative damage to cellular components but also regulate redox signaling in many cell types with essential functions in the cardiovascular system. Research over the past couple of decades has uncovered mechanisms by which NADPH oxidase (NOX) enzymes regulate oxidative stress and compartmentalize intracellular signaling in endothelial cells, smooth muscle cells, macrophages, cardiomyocytes, fibroblasts, and other cell types. NOX2 and NOX4, for example, regulate distinct redox signaling mechanisms in cardiac myocytes pertinent to the onset and progression of cardiac hypertrophy and heart failure. Heart failure with preserved ejection fraction (HFpEF), which accounts for at least half of all heart failure cases and has few effective treatments to date, is classically associated with ventricular diastolic dysfunction, i.e., defects in ventricular relaxation and/or filling. However, HFpEF afflicts multiple organ systems and is associated with systemic pathologies including inflammation, oxidative stress, arterial stiffening, cardiac fibrosis, and renal, adipose tissue, and skeletal muscle dysfunction. Basic science studies and clinical data suggest a role for systemic and myocardial oxidative stress in HFpEF, and evidence from animal models demonstrates the critical functions of NOX enzymes in diastolic function and several HFpEF-associated comorbidities. Here, we discuss the roles of NOX enzymes in cardiovascular cells that are pertinent to the development and progression of diastolic dysfunction and HFpEF and outline potential clinical implications.

## 1. Introduction

### 1.1. Heart Failure with Preserved Ejection Fraction (HFpEF)

Heart failure with preserved ejection fraction (HFpEF) is a complex, heterogenous, and highly prevalent multiorgan syndrome commonly associated with advanced age, obesity, and/or hypertension [1,2]. There are more than 3 million patients with HFpEF in the United States alone [3] and the prevalence is increasing dramatically due to associated cardiovascular and noncardiovascular comorbidities, the advanced age of the population, and increased recognition and diagnosis [1,4,5]. HFpEF results in classical clinical symptoms of congestive heart failure, including fatigue, exercise intolerance, and peripheral and pulmonary edema (i.e., congestion), but without a reduction in ejection fraction, although more subtle systolic impairment can often be detected [1,6,7]. The prognosis for HFpEF remains poor, with a 5-year mortality rate in excess of 50%, similar to heart failure with reduced ejection fraction (HFrEF) [3,4,8]. A number of cardiovascular insults and chronic diseases, including hypertension, arrhythmia, chronic kidney disease, and various metabolic conditions such as obesity and diabetes mellitus are common comorbidities and predispose patients to HFpEF. Consequently, there has been a dramatic increase in cases worldwide [1,3].

Diastolic dysfunction, or the inability of the ventricles to effectively relax and fill with blood, is a key cause of heart failure in patients with HFpEF and results in abnormally high left ventricular filling pressures, despite the maintenance of adequate left ventricular ejection fraction [9,10,11,12,13,14]. Concentric cardiac remodeling and left ventricular hypertrophy are frequently but not always present in HFpEF and can contribute to cardiac maladaptation [1,15]. Cardiac fibrosis, the deposition of excess extracellular matrix components in the cardiac interstitium, often occurs in HFpEF, reduces the compliance of the myocardium, and is a major contributor to the deterioration of diastolic function [1,10,12,13,16]. Systemic inflammation [1,3,9,10,17,18] and oxidative stress [1,10,17,18,19,20] are common components of the molecular etiology of HFpEF. Although the majority of reactive oxygen species (ROS) produced results from the inflammation of the endothelium [17,21,22], elevated myocardial oxidative stress also occurs in animal models [17,23,24,25,26] and patients with HFpEF [17,19,25] (Table 1). Moreover, elevated levels of reactive oxidative metabolites in the circulation correlate with rehospitalization or death due to a heart failure-related event in HFpEF [27,28], suggesting roles for oxidative stress in HFpEF disease severity and outcomes (Table 1).

### 1.2. NADPH Oxidase (NOX) Enzymes

Nicotinamide adenine dinucleotide phosphate (NADPH) oxidase (NOX) enzymes control the regulated cellular production of ROS and have indispensable functions in cardiovascular physiology and disease [31,32,33,34,35,36,37,38,39] (Table 2). ROS are short-lived, diffusible gaseous molecules that, in addition to oxidatively damaging cellular constituents, can locally oxidize cysteine or methionine residues on proteins to modify activity, function, oligomerization, and/or localization that can have profound impacts on intracellular signaling (i.e., redox signaling), including pathways that are instrumental to the onset and progression of cardiac hypertrophy and failure [31,32,33,40,41,42,43,44]. Although substantial ROS production occurs as a byproduct of oxidative metabolism in mitochondria [45,46] and can also be generated by monoamine oxidases [47,48,49], xanthine oxidase [50], or the uncoupling of the catalytic cycle of cytochrome P450s [51] and nitric oxide synthases (NOS) [52,53] (Section 4.1), the dedicated function of NOX enzymes is ROS production [37]. Thus, NOXs are the primary source of regulated, enzymatic ROS production and are responsible for a significant portion of ROS generated in the cardiovascular system, inducing oxidative stress and redox signaling central to the progression of cardiovascular disease [36,37].

There are seven NOX family enzymes (NOX1-5 and DUOX1-2), which are multi-pass transmembrane proteins that enzymatically generate superoxide via the transfer of electrons to molecular oxygen [36,37,38]. NOX1-5 have six transmembrane domains, whereas DUOX1 and 2 have seven transmembrane domains and are not expressed in cardiovascular cells [36,37]. NOX5 is expressed in human cardiomyocytes and vascular cells but not in rodents [36,38,54,55] and is regulated by calcium, similar to DUOX1 and 2 [36,37,38]. NOX3 expression is largely restricted to embryonic development and the inner ear [36]. NOX isoforms 1–4 require association with the membrane-embedded p22^phox^ regulatory subunit for oxidase activity and protein stability [36,37,38]. NOX1 and NOX2 (also known as gp91^phox^) require the translocation of additional cytosolic subunits to the oxidase complex for activation: NOX1 requires association with the p47^phox^ or NOXO1 organizer subunit, p67^phox^ or NOXA1 activator subunit, and the active (GTP-bound) Rho family small GTPase, Ras-related C3 botulinum toxin substrate 1 (Rac1), whereas NOX2 necessitates the interaction of p47^phox^, p67^phox^, p40^phox^, and active Rac1 or Rac2 with the NOX2:p22^phox^ complex at the membrane to evoke superoxide production [36,37,38,56,57]. 

**Figure 1:**
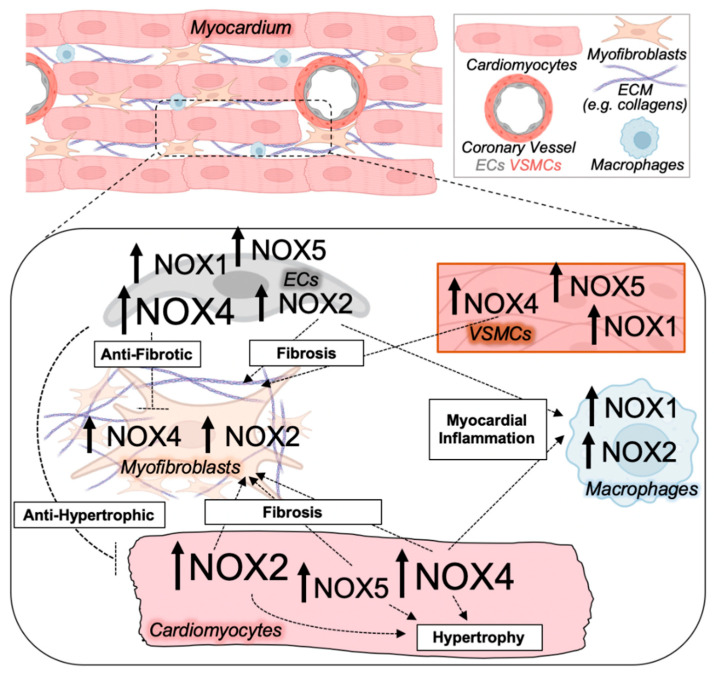
**NOX isoform upregulation and crosstalk between cell types in pathological cardiac remodeling.** The diseased myocardium is composed of several cell types all expressing NOX enzymes, including cardiomyocytes, activated myofibroblasts that deposit collagen and extracellular matrix (ECM) components, macrophages, and endothelial cells (ECs), as well as vascular smooth muscle cells (VSMCs) that make up the coronary vessels. NOX isoform activity and ROS production in various cell types of the heart mediate paracrine effects on other cardiac cell types to impact myocardial growth, inflammation, microvascular dysfunction, and fibrosis central to adverse cardiac remodeling. Magnitude and directionality of alterations in NOX isoform activity and/or expression in the indicated cardiac cell types are depicted by the font size and arrow, respectively.

**Figure 2:**
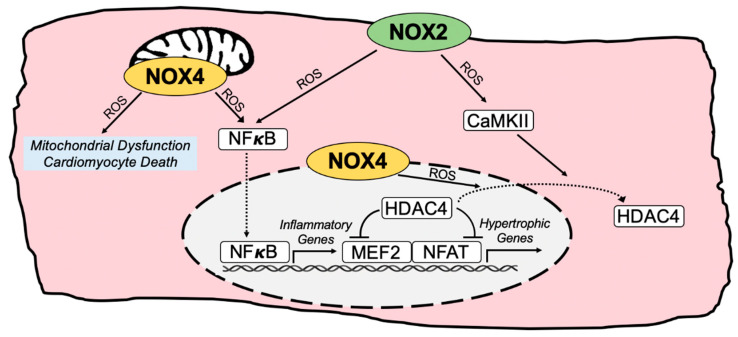
**Pathogenic NOX-regulated redox signaling circuitry in cardiac myocytes.** Depiction of major pathological redox-sensitive signaling effectors and pathways regulated by NOX2 and NOX4 in cardiomyocytes. *CaMKII*, calcium/calmodulin-dependent protein kinase II; *HDAC4*, histone deacetylase 4; *MEF2*, myocyte enhancer factor 2; *NFAT*, nuclear factor of activated T cells; *NFκB*, nuclear factor kappa B; *ROS*, reactive oxygen species.

With regards to the cardiovascular system, NOX1, 2, 4, and 5 are expressed in cardiomyocytes, endothelial cells, and vascular smooth muscle cells, and NOX2 and NOX4 in fibroblasts (Table 2, Figure 1). NOX2 is also robustly expressed in phagocytes, including neutrophils and macrophages [36,37,38,39,56,58,59], and NOX1 and NOX4 also function in monocytes and monocyte-derived macrophages [29,60]. These tissue- and cell type-specific expression patterns (Table 2, Figure 1), as well as distinct regulatory mechanisms governing NOX isoform enzyme activity and abundance, contribute to nonoverlapping redox-sensitive signaling circuitry and cellular functions that are regulated by different NOX isoforms (Figure 2).

NOX2 and NOX4 are the best-studied NOX enzymes in the cardiovascular system and cardiomyocytes more specifically. While the generation of ROS by NOX2 is inducible by Rac1/2 GTPase activity such as downstream of angiotensin receptor (AT1R) activation by angiotensin-II (AngII) [38,57,61], NOX4 exhibits constitutive activity but its levels are upregulated in response to pathological stimulation [38,62,63,64,65]. NOX4 is found at intracellular membrane organelles such as the endoplasmic reticulum [66,67], nuclear envelope [62], and mitochondria [35,63,65,67,68], whereas NOX2 is predominantly found at plasma membrane microdomains such as the T-tubule, lateral membrane, and intercalated disc in cardiomyocytes [69,70]. This results in the regulated compartmentalization of ROS production through the oxidase activity of distinct NOX isoforms. Cell type-specific expression patterns (Figure 1), the intracellular localization of NOX enzymes at distinct cell and organellar membranes (Figure 2), and unique regulatory mechanisms controlling NOX activity and expression enable the intricate spatiotemporal regulation of redox signaling through the dynamic modulation of NOX activity. Perhaps not surprisingly, NOXs have established roles in cardiac aging [32,35,68], cardiac hypertrophy [61,70,71], hypertension [72], atherosclerosis [34,38], diabetic cardiomyopathy [73], and myocardial infarction [40,58,74] (Table 2), so a significant role in HFpEF seems probable. 

## 2. NADPH Oxidases in Cardiovascular Disease

### 2.1. NOX Isoforms in Cardiac Hypertrophy, Fibrosis, and Diastolic Dysfunction

Given the paucity of animal models that faithfully recapitulate the multi-organ pathophysiology of HFpEF [1,3] and the lack of highly selective isoform-specific NOX inhibitors [37], there is limited knowledge on specific NOX enzymes in HFpEF. However, several studies have uncovered roles for NOX enzymes in diastolic dysfunction and the promotion of cardiac fibrosis in models of cardiac hypertrophy, injury, and failure (Table 2). NOX enzyme expression levels and/or oxidase activity are induced in cardiovascular cells in response to neurohumoral stimulation such as the activation of the renin-angiotensin-aldosterone system (RAAS) [36,39] that occurs in both HFrEF, as well as HFpEF [10,75,76,77,78], and thus, NOX-mediated oxidative stress and redox signaling in the heart and vasculature are likely to occur in HFpEF. Elevated levels of aldosterone or AngII in the circulation correlate with left ventricular hypertrophy and an increased risk of rehospitalization and mortality in patients with HFpEF [78,79,80,81], suggesting RAAS activation as a key pathomechanism in HFpEF. Notably, the pharmacological inhibition of NOX enzymes with apocynin reduces myocardial ROS production and diastolic dysfunction in rodent models of diabetes mellitus [82,83] and AngII infusion [84], as well as attenuating cardiac hypertrophy, fibrotic remodeling, and oxidative stress in response to AngII or aldosterone [84,85] and myocardial infarction injury [86], underscoring the centrality of NOXs to diastolic impairment and the deterioration of cardiac function in response to bolstered RAAS activity and comorbid cardiovascular disease. 

Basic science studies implicate NOX2, NOX4, and NOX5 in the deterioration of diastolic function and adverse cardiac remodeling in response to pathological stimuli (Table 2). A global loss of Nox2 limits myocardial ROS production and preserves diastolic function in response to diet-induced obesity [87], doxorubicin treatment [88], and myocardial infarction [89], and mitigates cardiac fibrosis in response to AngII, aldosterone, or pressure overload [41,90,91,92], whereas Nox2 overexpression in endothelial cells promotes cardiac fibrosis, inflammation, and diastolic dysfunction in response to AngII [93]. Inducible NOX2 activity in the heart in response to AngII appears to be particularly important for adverse cardiac remodeling and oxidative stress, as the loss of Nox2 protects from AngII-induced myocardial oxidative stress, hypertrophy, and fibrosis without altering AngII-induced hypertension [41]. Similarly, the cardiomyocyte-specific loss of Rac1, which is required for the induction of NOX2 oxidase activity, rescues diastolic dysfunction in response to pressure overload [94] and suppresses cardiac hypertrophy, fibrosis, and oxidative stress in response to AngII [61] and streptozotocin-induced diabetes [83]. Moreover, statin treatment, which represses small GTPase signaling by inhibiting the biosynthesis of lipid precursors needed for protein prenylation [95], impairs myocardial Rac1 activation and oxidative stress in rats in response to AngII or pressure overload [96] and in human heart failure [97]. Statin treatment is also associated with reduced cardiomyocyte hypertrophy and resting tension in myocardium from patients with HFpEF [22], suggesting that an effective in vivo strategy to target NOX2 could alleviate cardiac maladaptation in HFpEF. 

Many studies have also revealed indispensable functions of NOX4 in cardiac biology and disease. The transgenic overexpression of Nox4 at modest levels in cardiomyocytes elicits elevated myocardial ROS production, fibrosis, and cardiomyocyte hypertrophy and apoptosis with advanced age [63]. The cardiomyocyte-specific overexpression of human NOX4, at higher levels that recapitulate upregulation in response to AngII, was sufficient to induce myocardial ROS and fibrosis comparable to AngII treatment, but in the absence of cardiac hypertrophy [98]. Moreover, NOX4 overexpression further promoted cardiac hypertrophy, fibrosis, and oxidative stress in response to AngII [98]. The role of NOX4 in cardiac pressure overload is somewhat unclear, with one study reporting protection by the cardiomyocyte-specific deletion of Nox4, while another study found that the deletion of Nox4 in cardiomyocytes or endothelial cells exacerbated pressure overload-induced cardiac hypertrophy, dysfunction, and fibrosis [99,100]. Consistent with a role for cardiomyocyte NOX4 in the maladaptation of the heart to RAAS hyperactivation, the treatment of mice with a mitochondrial-targeted antioxidant peptide prevented the upregulation of Nox4 and mitochondrial oxidative stress, as well as ameliorated cardiac hypertrophy, fibrosis, and diastolic dysfunction in response to AngII [64]. 

NOX4 also has critical roles in the inflammatory and wound healing response of the heart to ischemic injury. Although the transgenic overexpression of Nox4 in cardiac myocytes was sufficient to promote macrophage recruitment to the heart and polarization towards the M2 phenotype that was associated with a modest reduction in cardiac hypertrophy, fibrosis, and mortality following myocardial infarction [101], endogenous Nox4 plays an indispensable role in cardiac inflammation and tissue damage in the infarcted heart, as mice with a global loss of Nox4 exhibited less myocardial infarction-induced macrophage infiltration, ischemic injury, cardiomyocyte hypertrophy, and oxidative stress [74]. Moreover, the overexpression of a dominant-negative Nox4 mutant resulted in reductive stress and increased mitochondrial ROS, infarction size, and myocardial energetic deficits in response to ischemia-reperfusion injury [102], suggesting that NOX4 also has adaptive functions in modulating inflammation and injury resolution in the ischemic myocardium. Since advanced age is a major risk factor for the development of HFpEF [2,103] and oxidative stress is a major contributor to the aging of the heart and other organs [32,68,104], it is notable that Nox4 protein levels increased with age in the heart [63] and treatment with a pharmacological NOX1/NOX4 inhibitor preserved cardiac function in aged mice [68]. More importantly perhaps, cardiac Nox4 protein levels were upregulated in a mouse model of HFpEF [30]. It is also noteworthy that NOX2 and NOX4 contribute to atrial oxidative stress and susceptibility to atrial fibrillation [54,105,106,107,108,109,110,111,112], a comorbidity that is present in roughly one third of patients with HFpEF that can result in chronic arrhythmogenicity that precipitates the progression of cardiac failure [113,114,115,116,117].

Although not expressed in rodents, gain-of-function studies in mice suggest a role for the calcium-sensitive NOX5 enzyme in the adverse remodeling of the heart in response to RAAS activation or mechanical stress. Indeed, the cardiomyocyte-specific overexpression of human NOX5 in mice exacerbated cardiac hypertrophy, fibrosis, and oxidative stress in response to AngII infusion and cardiac pressure overload [55]. Thus, NOX enzymes have well-established physiologic functions in various cell types of the cardiovascular system [31,36,37], many of which are likely important in the context of HFpEF. Here, we discuss evidence for regulated ROS production by NOX enzymes in diastolic dysfunction and HFpEF and the NOX-regulated intracellular hypertrophic signaling mechanisms in cardiomyocytes controlled by NOX2 and NOX4.

### 2.2. NADPH Oxidases in Non-Myocyte Cardiac Cell Types

NOX enzymes are expressed in all cell types of the vasculature including the coronary vessels supplying blood to the heart [36,37,38]. Vascular NOX-generated ROS play essential roles in cardiovascular oxidative stress, aging, atherosclerosis, angiogenesis, inflammation, and blood pressure homeostasis [36,37,118,119,120,121]. Crosstalk between NOX enzymes and redox signaling pathways in the coronary vasculature, fibroblasts, and immune cells of the heart are likely involved in coronary microvascular dysfunction, inflammation, and myocardial relaxation defects in HFpEF (Figure 1).

#### 2.2.1. NOX Enzymes in the Vasculature

NOX4 is the most abundant NOX isoform in endothelial cells and a major source of ROS production in the vasculature and heart [37,122]. In contrast to the maladaptive roles of most ROS generated by NOX enzymes in the cardiovascular system, endothelial NOX4 is protective (Table 2). The overexpression of Nox4 in endothelial cells reduces hypertension, myocardial inflammation, and fibrosis in response to AngII [118,123], whereas the endothelial cell-specific loss of Nox4 exacerbates cardiac hypertrophy, dysfunction, and fibrosis in response to pressure overload [100]. Similarly, the endothelial-specific overexpression of Nox4 attenuates atherosclerotic remodeling and vascular inflammation in ApoE^−/−^ mice [124], while the loss of Nox4 promotes the formation of atherosclerotic lesions and vascular inflammation [125].

**Figure 3:**
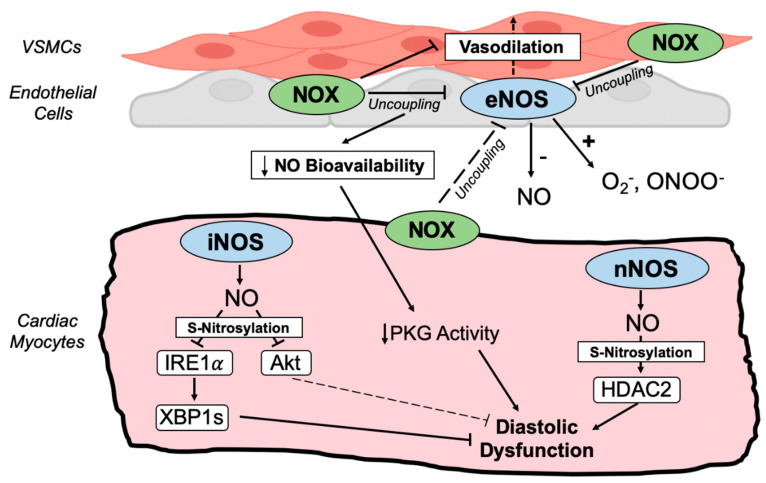
**Crosstalk between NADPH oxidases and nitric oxide synthases.** Schematic depiction of redox-nitroso crosstalk between coronary vasculature and cardiac myocytes in HFpEF. Endothelial NOS (eNOS) promotes vasodilation that is impaired when oxidative stress uncouples eNOS, diminishing NO bioavailability, cGMP levels, and protein kinase G (PKG) activity. Decreased myocardial PKG activity facilitates diastolic dysfunction by reducing titin phosphorylation. Uncoupled NOS enzymes generate damaging superoxide and peroxynitrite that further promote oxidative stress. Nitrosative stress in the HFpEF myocardium, on the other hand, is provoked by the upregulation of inducible NOS (iNOS) and neuronal NOS (nNOS) in cardiac myocytes. nNOS-dependent S-nitrosylation of histone deacetylase 2 (HDAC2) and defective XBP1 splicing (XBP1s) due to iNOS-dependent S-nitrosylation of IRE1𝛼 promote diastolic dysfunction and heart failure in HFpEF animal models. iNOS also mediates Akt S-nitrosylation. NOS uncoupling and nitrosative stress-responsive signaling contribute to oxidative stress, coronary microvascular endothelial dysfunction, and impaired myocardial relaxation in HFpEF.

Conversely, endothelial NOX1, NOX2, and NOX5 contribute to impaired vascular function, endothelial nitric oxide synthase (eNOS) uncoupling (Figure 3), and adverse vascular remodeling and hypertension [38,126]. Indeed, endothelial cell Nox1 is required for hypertension in response to AngII [127], while NOX2 overexpression in endothelial cells further promotes blood pressure elevation in response to AngII [128] and stimulates macrophage recruitment and the initiation of atherosclerotic plaque formation in ApoE^−/−^ mice [129]. Most notable with regards to HFpEF pathophysiology, endothelial cell NOX2 is instrumental to inflammatory cell recruitment, myocardial fibrosis, and diastolic dysfunction in response to AngII [93]. The expression of NOX5 is increased in the coronary arteries of atherosclerosis patients [130], and the endothelial cell-specific expression of human NOX5 in mice results in hypertension with advanced age via the uncoupling of eNOS (Figure 3) and the repression of cyclic GMP (cGMP) signaling [131], indicating maladaptive roles for endothelial NOX5 in cardiovascular disease.

NOX1, NOX4, and NOX5, and to a lesser extent NOX2, are expressed in vascular smooth muscle cells [39,126,132]. Nox1 overexpression in vascular smooth muscle cells increases vascular ROS production, blood pressure, aortic medial thickness, and the uncoupling of eNOS in response to AngII [133,134] while a deficiency of NOX1 limits neointima formation in response to wire injury [135] and vascular smooth muscle cell migration in response to AngII or platelet-derived growth factor (PDGF) [135,136]. Moreover, Noxa1, the activator subunit for Nox1, in vascular smooth muscle cells is indispensable for fulminant atherosclerotic remodeling and oxidative stress in multiple models of dyslipidemia [34].

NOX4 in vascular smooth muscle cells plays critical roles in vascular aging, inflammation, and atherogenesis. NOX4 expression is upregulated in the atherosclerotic lesions of mouse models and humans [137,138] and is required for ROS generation and the induction of an inflammatory gene expression program in vascular smooth muscle cells in response to transforming growth factor-β (TGF-β) [137]. NOX4 in the mitochondria of vascular smooth muscle cells is particularly important in cardiovascular disease. The overexpression of mitochondrial-targeted Nox4 elicited a vascular aging phenotype including aortic stiffening, fibrosis, elevated mitochondrial ROS production, and the impairment of aortic smooth muscle cell contractility and mitochondrial respiration [35]. In contrast, the knockdown of Nox4 in aortic smooth muscle cells from aged mice reduced mitochondrial ROS generation and improved mitochondrial function [68].

The calcium-dependent NOX5 enzyme is upregulated in the vascular smooth muscle cells of hypertensive patients and is required for AngII- and PDGF-induced ROS production [139,140]. NOX5 promotes vascular contractility, as the transgenic expression of human NOX5 in mouse vascular smooth muscle cells inhibits endothelium-dependent relaxation and increases vasoconstriction in isolated mesenteric arteries [139,141]. NOX5 also incites vascular calcification in response to calcium through its oxidase activity in vascular smooth muscle cells [142]. NOX enzymes in the coronary and peripheral vasculature contribute to systemic oxidative stress, inflammation, and vascular remodeling, all of which facilitate the development of HFpEF. It is plausible that the well-established functions of NOX-generated ROS in vascular oxidative stress, remodeling, and redox signaling also contribute to myocardial intercellular communication and microvascular inflammation in HFpEF.

#### 2.2.2. NOX Enzymes in Fibroblasts

Cardiac fibrosis is a major driver of cardiac pathology and diastolic dysfunction in HFpEF [10,12]. Resident cardiac fibroblasts in the injured heart transform into myofibroblasts that synthesize and secrete extracellular matrix proteins such as collagens, thereby promoting wound healing but impinging upon myocardial elasticity [3,10,143]. Unfortunately, studies of NOX enzymes specifically in cardiac fibroblasts remain limited. NOX enzymes in vascular adventitial fibroblasts play vital roles in vascular inflammation and signaling to other vascular cell types [144]. Fibroblast Nox2 is required for AngII-induced hypertension and vascular remodeling and mediates paracrine signaling through the secretion of growth differentiation factor 6 (GDF6), which promotes vascular smooth muscle cell growth [59].

NOX4 is the best-studied NOX isoform in cardiac fibroblasts and has important roles in the fibrotic remodeling of the heart. NOX4 is upregulated in cardiac fibroblasts in response to TGF-β and in heart failure and is required for TGF-β-induced superoxide production, Smad2/3 phosphorylation, myofibroblast transformation, and collagen production [145,146]. Future studies using Cre lines to temporally control the deletion of NOX isoforms in cardiac fibroblast lineages will help to delineate the functions of NOX enzymes in cardiac fibroblasts, fibrotic remodeling, and the stiffening of the ventricular myocardium.

#### 2.2.3. NOX Enzymes in Immune Cells

NADPH-dependent oxidase activity was originally discovered in neutrophils and was later found to be mediated by NOX2 [56,147,148,149]. Indeed, NOX2 is robustly expressed in phagocytes, including not only neutrophils, but also macrophages [150] that infiltrate the stressed heart to promote wound healing and injury resolution, particularly in response to myocardial infarction [151,152]. The recruitment of monocyte-derived macrophages in nonischemic heart failure is also a critical component of disease progression, fibrotic remodeling, and decompensation [153,154]. NOX2 levels are increased in cardiac macrophages and peripheral monocytes in human patients with HFpEF and the obese ZSF1 rat model of HFpEF [17,29]. Nonetheless, a direct role for NOX2 in resident cardiac macrophages or monocyte-derived macrophages recruited to the injured heart has not been directly examined.

NOX4 generates ROS in peripheral monocytes and macrophages where it is upregulated in response to oxidized low-density lipoprotein to promote macrophage cytotoxicity [60], suggesting roles for monocyte NOX4 in atherosclerosis; yet similarly, no studies have interrogated NOX4 in cardiac macrophages to date. It is noteworthy, however, that NOX4 in cardiomyocytes has crucial roles in the recruitment of macrophages to the heart in response to ischemic injury [101]. Perhaps most germane to the molecular etiology of HFpEF, the global loss of Nox1 mitigates cardiac hypertrophy, coronary endothelial cell activation, and macrophage recruitment in a mouse model of metabolic cardiomyopathy [29]. Intriguingly, the expression levels of *NOX1* in peripheral monocytes correlate with the degree of diastolic dysfunction in patients with HFpEF [29], indicating roles for monocyte NOX1 in inflammation and defective myocardial relaxation in HFpEF.

NOX-dependent superoxide production by peripheral blood mononuclear cells is increased in patients with hypertension compared to normotensive controls and even further enhanced among patients with hypertension and left ventricular hypertrophy [155], suggesting critical roles for NOX enzymes in lymphocytes and monocytes in hypertension and cardiac hypertrophy. Recent studies elucidated required functions for NOX2 in regulatory T cells (Tregs) that suppress inflammatory responses in cardiac and vascular remodeling. The Treg-specific deletion of Nox2 or the adoptive transfer of Tregs lacking Nox2 reduced blood pressure, cardiac hypertrophy, and cardiac fibrosis in response to AngII through a mechanism that involved increasing the number of cardiac resident Tregs and promoting their immune suppressive activity [156]. Thus, NOXs in immune cells likely contribute directly to both systemic and local inflammatory responses in the heart in HFpEF and other forms of heart disease. Analyses of NOX activity in peripheral blood cells in human patients with HFpEF coupled with studies using HFpEF animal models and the genetic manipulation of NOX expression will help to reveal the cell type-specific functions of NOX isoforms in diastolic dysfunction and cardiac failure.

## 3. NOX-Regulated Pathogenic Redox Signaling in Cardiomyocytes

NOX2 and NOX4 have emerged as central regulators of the intracellular redox signaling landscape in cardiomyocytes (Figure 2). NOX2 is activated by pathological stimuli such as AngII, aldosterone, and pressure overload [37,57,71], enabling the rapid inducibility of superoxide production [37,57,71,91]. In contrast, NOX4 has constitutive basal oxidase activity but its expression levels are upregulated in the heart by cardiovascular stress, such as in response to AngII, adrenergic stimulation, pressure overload [38,62,63,64,65], or the high-fat diet and N^ω^-nitro-L-arginine (HFD/L-NAME) model of HFpEF [30]. NOX4 is predominantly localized at intracellular organelle membranes, including at mitochondria, the endoplasmic reticulum, and nuclear envelope [62,63,67,157]. Thus, the positioning and activity of NOX2 at sarcolemmal membrane domains and NOX4 at intracellular membranes dictate the topography of redox signaling that can have instrumental roles in cardiac hypertrophy and diastolic dysfunction (Figure 2).

### 3.1. Redox Regulation of Hypertrophic and Maladaptive Gene Expression Programs

Notably, both NOX2 and NOX4 activity promote the activation of hypertrophic gene expression in cardiomyocytes through distinct mechanisms that provoke oxidation-dependent nuclear export of histone deacetylase 4 (HDAC4), thereby derepressing the transcriptional activity of the hypertrophic transcription factors, myocyte enhancer factor 2 (MEF2) and nuclear factor of activated T cells (NFAT) [40,42,44,62]. The NOX2-dependent oxidation of calcium/calmodulin-dependent protein kinase II (CaMKII) in response to AngII or aldosterone results in the noncanonical activation of CaMKII and the consequent phosphorylation and nuclear export of HDAC4 that induces MEF2-dependent transcription and cardiomyocyte hypertrophy [40,44] (Figure 2). In contrast, NOX4 at the nuclear envelope induces the direct oxidation of HDAC4 in response to phenylephrine that similarly promotes the nuclear-to-cytoplasmic shuttling of HDAC4, thereby activating pro-hypertrophic NFAT-dependent transcription [42,62] (Figure 2). 

Nuclear factor kappa B (NFκB) transcriptional activity regulates inflammatory gene expression programs and is enhanced in a NOX2-dependent manner in response to AngII [61,158]. Indeed, Rac1 in cardiomyocytes is required for oxidative stress and NFκB activation in response to AngII [61], while Nox4 overexpression in cardiomyocytes is also sufficient to induce NFκB activation [98], suggesting roles for NOX2 and NOX4 in the regulation of NFκB signaling and inflammatory gene expression that may contribute to myocardial remodeling in HFpEF (Figure 2). Thus, NOX2 and NOX4 control unique redox-sensitive hypertrophic signal transduction circuitry that can integrate multiple inputs and converge on the regulation of maladaptive gene expression programs.

### 3.2. Sarcoplasmic Reticulum Calcium Handling and Myocardial Contractility 

NOX2 activity in cardiomyocytes promotes sarcoplasmic reticulum calcium cycling and contractility in response to AngII in part through the hyperphosphorylation of phospholamban [70], while mechanical stretch evokes the NOX2-dependent sensitization of ryanodine receptors and arrhythmogenic calcium sparks [69], both of which could also be enhanced by the NOX2-mediated oxidation of CaMKII. 

### 3.3. Mitochondrial ROS and Cardiomyocyte Death

NOX4-generated ROS at cardiac mitochondria can influence redox signaling and promote mitochondrial oxidative damage and cardiomyocyte death [63,64] (Figure 2). Nox4 overexpression is sufficient to induce mitochondrial dysfunction and cardiomyocyte apoptosis with aging [63] and Nox4 in cardiomyocytes is required for the pressure overload-induced production of mitochondrial ROS and cardiomyocyte apoptosis [99]. Moreover, AngII infusion in mice promotes Nox4 expression in conjunction with mitochondrial oxidative damage, diastolic dysfunction, fibrosis, and apoptosis, all of which are normalized by the administration of a mitochondrial-targeted antioxidant peptide [64]. Thus, the targeted inhibition of mitochondrial NOX4 could potentially exert therapeutic benefits in patients with diastolic dysfunction and/or HFpEF by limiting excessive mitochondrial ROS production (Figure 2). Notably, however, the broad inhibition of NOX4 could repress its adaptive functions in response to cardiomyocyte stress, including at mitochondria-associated membranes and the endoplasmic reticulum, where it inhibits mitochondrial calcium overload [67] and induces autophagy in response to nutrient deprivation [66], respectively.

## 4. NADPH Oxidases and Nitric Oxide (NO) Signaling

### 4.1. Nitric Oxide Synthase (NOS) Uncoupling and cGMP/Protein Kinase G (PKG) Signaling

Nitric oxide (NO), which is predominantly generated in the endothelium by eNOS, has potent vasodilatory activity in the coronary and peripheral vasculature via the activation of soluble guanylate cyclase (sGC) in vascular smooth muscle cells [159,160] (Figure 3). NOX-generated ROS interfere with the catalytic cycle of NOS enzymes, resulting in the production of superoxide rather than NO that reduces NO bioavailability, impairing cGMP-dependent vascular smooth muscle relaxation, protein kinase G (PKG) signaling, and further contributing to oxidative stress [21,36,161]. Additionally, the uncoupling of NOS enzymes impairs myofilament relaxation and diastolic function by dampening the PKG-mediated phosphorylation of titin [21,36,162,163]. Peroxynitrite is also generated from NO and superoxide by uncoupled NOS enzymes, further reducing NO bioavailability and resulting in protein nitration by the reaction of peroxynitrite with tyrosine residues [164] (Figure 3). Indeed, myocardial cGMP levels and PKG activity are substantially reduced in the myocardium of patients with HFpEF [165], as are the serum levels of NO-derived metabolites [166,167], whereas myocardial nitrotyrosine levels are elevated [165], suggesting that NOS uncoupling and oxidative stress contribute markedly to HFpEF pathophysiology. Thus, NOX-generated ROS can facilitate NOS uncoupling that both attenuates the cardiovascular protection afforded by NO and further promotes the generation of damaging ROS and reactive nitrogen species [21,36].

### 4.2. Nitrosative Stress and Protein S-Nitrosylation

Despite the elevation of ROS and the dampening of signaling downstream of NO (i.e., cGMP levels, PKG activity) in HFpEF, paradoxically, NO and nitrosative stress seem to be nodal drivers of cardiac disease in HFpEF (Figure 3). NO can be covalently attached to protein cysteines, termed S-nitrosylation, which serves as a post-translational mechanism that can modulate protein activity and function [159,168]. Indeed, multiple studies have uncovered pathogenic roles for NO in HFpEF, mediated at least in part through protein S-nitrosylation that is upregulated in the myocardium of mouse models and human patients with HFpEF [7,169]. Most notably, inducible NOS (iNOS) is upregulated in cardiomyocytes and is required for cardiac hypertrophy, fibrosis, diastolic dysfunction, edema, mitochondrial abnormalities, and exercise intolerance in the HFD/L-NAME mouse model of HFpEF [7,30]. S-nitrosylation of the endoplasmic reticulum stress transducing protein, inositol-requiring enzyme 1𝛼 (IRE1𝛼), is induced in HFpEF and prevents the IRE1𝛼 -mediated mRNA splicing of its effector, *X-box-binding protein 1* (*Xbp1*), that is required for the ER stress response and cardiac adaptation [7,170]. Importantly, the loss of iNOS or cardiomyocyte-specific overexpression of spliced Xbp1 rescues cardiac and systemic HFpEF phenotypes in the HFD/L-NAME model [7], suggesting essential roles for the iNOS-dependent S-nitrosylation of IRE1𝛼 in HFpEF. Akt S-nitrosylation at Cys-224 is also induced by iNOS in response to HFD/L-NAME, resulting in the inhibition of cardioprotective Akt signaling by reducing Ser-473 phosphorylation [30]. Thus, the upregulation of iNOS in HFpEF cardiomyocytes initiates pathologic S-nitrosylation-regulated signaling.

S-nitrosylation of histone deacetylase 2 (HDAC2) by neuronal NOS (nNOS) also plays a key role in HFpEF pathophysiology [169]. nNOS expression and HDAC2 S-nitrosylation are elevated in the hearts of patients with HFpEF and mice subjected to the salty drinking water/unilateral nephrectomy/aldosterone (SAUNA) model of HFpEF [169]. The genetic ablation of nNOS or knock-in mutation of the HDAC2 S-nitrosylation sites (Cys262/274) mitigates SAUNA-induced HDAC2 S-nitrosylation, diastolic dysfunction, and exercise exhaustion [169]. Thus, the upregulation of iNOS and nNOS in HFpEF facilitates the activation of nitrosative stress-responsive signaling pathways that are critical for the development of cardiac and systemic pathologies in HFpEF animal models and are a distinguishing feature of HFpEF versus HFrEF (Figure 3).

The interplay between oxidative stress and NO production is complex but basic science studies and clinical data suggest that both NOX and NOS enzymes and oxidative and nitrosative stress play important roles in HFpEF. The NOX-mediated uncoupling of NOS enzymes in the vasculature could limit NO production and further promote the generation of ROS and reactive nitrogen species, oxidative tissue damage, and pathogenic redox signaling [21,36,161]. However, enhanced nitrosative stress is emerging as a hallmark and defining feature of HFpEF myocardium that occurs largely as a consequence of the induction of iNOS and nNOS levels and activity in cardiac myocytes [1,7,169]. There is also evidence that nitrosative stress can promote NOX activity and redox signaling in HFpEF, as iNOS was required for the upregulation of cardiac Nox4 levels and mitochondrial oxidative stress in response to HFD/L-NAME [30]. Further investigation of NOX and NOS isoforms in diastolic dysfunction will shed light on nitroso-redox crosstalk and redox/nitrosative signaling mechanisms that participate in the onset and progression of HFpEF.

## 5. Clinical Implications and Future Perspectives

### 5.1. Renin-Angiotensin-Aldosterone System (RAAS) Antagonists

There is interest in targeting oxidative stress, and NOX isoforms in particular, as therapeutic avenues to delay adverse outcomes in HFpEF. As discussed above, NOXs play essential roles in cardiovascular oxidative stress and redox signaling in response to RAAS activation. NOX2- and NOX4-dependent ROS generation are specifically induced in the heart in response to elevated AngII, aldosterone, and catecholamines in the circulation in cardiovascular disease and can contribute to the deterioration of diastolic function. Unfortunately, for the most part, pharmacological strategies used to treat HFrEF have not been effective at reducing mortality or cardiovascular events in patients with HFpEF [1,2,171]. Although serum aldosterone levels correlate with the risk of rehospitalization and death in HFpEF [79], mineralocorticoid receptor antagonist (MRA) treatment did not reduce the risk of death or heart failure rehospitalization in HFpEF [172]. These data suggest that the inhibition of NOX2 activity downstream of the mineralocorticoid receptor with MRA therapy is insufficient to substantially alter outcomes in patients with HFpEF. Similarly, angiotensin receptor blocker (ARB) [173], angiotensin converting enzyme (ACE) inhibitor [174,175], or ARB/neprilysin inhibitor (sacubitril) [176] therapy have had insufficient efficacy in the treatment of HFpEF, indicating that antagonism of the RAAS system alone, at least with existing pharmacologic agents and the timing of therapeutic intervention, does not significantly impede HFpEF disease progression. It is possible that some NOX activation mechanisms in HFpEF (e.g., the upregulation of NOX4 expression levels [30,62,63]) could evade RAAS inhibition, or that NOX-regulated oxidative damage and/or activation of pathogenic redox signaling prior to treatment cause enduring adverse effects on cardiac remodeling, such as through the activation of prohypertrophic transcriptional pathways (Figure 2, Section 3.1). Further exploration of strategies targeting RAAS activity, their antioxidant properties, and efficacy in combination therapies in HFpEF is warranted.

### 5.2. Nitric Oxide Donors and cGMP/Protein Kinase G Signaling

Similar to commonly prescribed heart failure therapies targeting the RAAS pathway, nitric oxide donor therapies have failed to improve HFpEF outcomes [177,178]. Neither isosorbide mononitrate [177] nor inorganic nitrite [178] had an impact on diastolic function, exercise capacity, or the risk of adverse cardiovascular events in patients with HFpEF. Nitrates may circumvent some pathogenic consequences of reduced NO bioavailability in HfpEF, but the induction of pathogenic NOX-regulated redox signaling mechanisms may not be perturbed. Nitrates could promote the elevation of myocardial cGMP levels and PKG activity that are reduced in HFpEF [17,165], but signaling activated by NOX enzymes in the heart (Figure 2) in response to increased circulating aldosterone, AngII, and enhanced sympathetic activity in HFpEF may remain unchanged. Targeting cGMP/PKG signaling with a soluble guanylate cyclase activator (vericiguat) [179], phosphodiesterase-5 inhibitor (sildenafil) [180], or neprilysin inhibitor (sacubitril) [176] was similarly not effective in HFpEF clinical trials.

Another important caveat to NO donor therapy is that it could even promote pathogenic nitrosative stress in the heart and the upregulation of S-nitrosylation-dependent signaling mechanisms in cardiomyocytes that are central to HFpEF disease progression [7,169] (Figure 3, Section 4.2). Indeed, IRE1α and HDAC2 S-nitrosylation are required for diastolic dysfunction in pre-clinical models of HFpEF [7,169], and these pathways may be further activated by nitrates.

### 5.3. Sodium-Glucose Cotransporter 2 Inhibitors

Excitingly, recent clinical trials have demonstrated that sodium-glucose cotransporter 2 (SGLT2) inhibitors, which lower blood glucose by repressing glucose resorption in the proximal tubule of the kidney [181,182], reduce the risk of death or hospitalization and improve exercise capacity and heart failure symptoms in patients with HFpEF [183,184]. The success of SGLT2 inhibitors in the treatment of HFpEF is a breakthrough in heart failure therapeutics that underscores the systemic and cardiometabolic nature of HFpEF. Although originally designed for the treatment of diabetes, SGLT2 inhibitors have proven effective in treating HFrEF [185,186] and HFpEF [183,184] in patients both with and without diabetes. SGLT2 inhibitors have obvious benefits on blood glucose homeostasis and cardiac bioenergetics [181,182], but their marked success independent of diabetes as a comorbidity has pointed to additional mechanisms of cardioprotection, including antioxidant and anti-inflammatory effects on the heart [25,187]. Most notably, empagliflozin attenuates myocardial oxidative stress in human HFpEF [188], the ZDF obese rat HFpEF model [25], and multiple mouse models of diabetic cardiomyopathy [189,190]. The amelioration of myocardial redox status in response to empagliflozin is associated with the restoration of NOS coupling, PKG-mediated titin phosphorylation, and diastolic function [25]. Intriguingly, cardiac Nox4 protein levels are elevated in the diabetic rodent heart, an effect that is normalized by empagliflozin treatment along with myocardial oxidative stress, fibrosis, and cGMP levels [189,190]. Canagliflozin similarly blunted the upregulation of Nox4, as well as the induction of iNOS protein levels in the mouse heart in response to β-adrenergic stimulation [191], collectively suggesting that a reduction in NOX4-generated ROS and nitrosative stress may contribute to the efficacy of SGLT2 inhibitors in the treatment of HFpEF. Canagliflozin also reduced Rac1 activation, membrane translocation, NOX activity, and ROS levels in cultured ex vivo atrial myocardium [192], suggesting that SGLT2 inhibitors may also disrupt NOX2-dependent oxidative stress and/or redox signaling. Thus, impacts on NOX2, NOX4, and myocardial nitroso-redox status may be indirect beneficial effects of SGLT2 inhibitor therapy in HFpEF.

### 5.4. Future Perspectives

Future studies interrogating the regulation of NOX-mediated oxidative stress and redox signaling by distinct NOX isoforms in various cell types of the heart using pre-clinical models of HFpEF and diastolic dysfunction will facilitate the identification of novel targets to repress oxidant stress and pathogenic redox signaling. An examination of the phenotypic response, myocardial redox status, and redox signaling landscape of NOX isoform-specific conditional deletion mice in models of HFpEF (e.g., HFD/L-NAME, SAUNA) will help to uncover the mechanistic contribution of NOXs to HFpEF. Evidence from humans and animal models suggests that NOX-regulated oxidative stress and redox signaling mechanisms likely participate in HFpEF pathogenesis. Further investigation could suggest the development of isoform-specific NOX inhibitors, the targeting of regulators of NOX oxidase activity (e.g., Rac1), or strategies to intervene with specific NOX-regulated redox signaling effectors as therapeutic targets for the treatment of diastolic dysfunction and HFpEF.

## Figures and Tables

**Table 1:** Reported evidence of oxidative stress and dysregulation of NOX enzymes in patients and animal models with HFpEF.

Patients with HFpEF	Effect
Myocardium	• Trend towards increased NOX2 expression in cardiac macrophages [17] • Increased myocardial H_2_O_2_ levels [17,25] • Increased myocardial lipid peroxidation [25]
Serum	• Elevated levels of derivatives of reactive oxidative metabolites (DROMs) in patients with HFpEF with HF-related events [27,28] • Increased thiobarbituric acid reactive substances (TBARS, biomarker of lipid peroxidation) [19]
Peripheral Blood Monocytes	• Increased *NOX1* and *NOX4* mRNA levels correlate with diastolic dysfunction in patients with HFpEF [29]
**Pre-clinical HFpEF Models**	**Effect**
HFD ^†^/L-NAME (mice)	• Increased myocardial Nox4 protein expression [30]
Unilateral nephrectomy and aldosterone infusion (mice)	• Increased myocardial oxidative stress (DHE fluorescence) [23,24]
Obese ZSF1/ZDF rats	• Increased cardiac macrophage Nox2 [17] • Increased myocardial H_2_O_2_ levels [17,25] • Increased myocardial lipid peroxidation [25]
DOCA/Western diet ^††^ (Göttingen miniswine)	• Increased 8-isoprostane levels in plasma [20]
Streptozotocin, HFD ^†††^, and renal artery embolization (Yorkshire x Landrace swine)	• Increased myocardial NADPH-stimulated superoxide production [26]

*DHE*, dihydroethidium (ROS sensor); DOCA, 11-deoxycorticosterone acetate (mineralocorticoid/glucocorticoid); *DROMs*, derivatives of reactive oxidative metabolites; *HFD*, high-fat diet; *HFpEF*, heart failure with preserved ejection fraction; *L-NAME*, N^ω^-nitro-L-arginine (NOS inhibitor); *NADPH*, nicotinamide adenine dinucleotide phosphate; *TBARS*, thiobarbituric acid reactive substances. Diet information: ^†^ 60% of calories from lard; ^††^ 1% cholesterol, 20% fat, 8.9% fructose, 2% salt; ^†††^ 10% sucrose, 15% fructose, 25% saturated fats, 1% cholesterol.

**Table 2:** **Cell type distribution of NOX isoforms and functions in cardiac and vascular pathologies.** “+” indicates promotion of the designated cardiovascular disease phenotype, whereas “−” indicates repression of the disease phenotype. *CMs*, cardiomyocytes; *ECs*, endothelial cells; *VSMCs*, vascular smooth muscle cells. See Section 2.

NOX Isoform	Cell Types	Cardiac			Vascular	
		Hypertrophy	Fibrosis	Inflammation	Hypertension	Atherosclerosis
**NOX1**	CMs, ECs, VSMCs, Macrophages	+	+	+	+	+
**NOX2**	CMs, ECs, VSMCs, Macrophages, Fibroblasts	+	+	+	+	+
**NOX4**	CMs, VSMCs, Macrophages, Fibroblasts	+	+	+		+
	ECs	−	−	−	−	−
**NOX5**	CMs, ECs, VSMCs	+	+		+	

## Abbreviations

ACE	angiotensin converting enzyme
AngII	angiotensin II
ARB	angiotensin receptor blocker
AT1R	angiotensin receptor type 1
CaMKII	calcium/calmodulin-dependent protein kinase II
cGMP	cyclic GMP
eNOS	endothelial nitric oxide synthase
GDF6	growth differentiation factor 6
HDAC2, 4	histone deacetylase 2, 4
HFD	high-fat diet
HFpEF	heart failure with preserved ejection fraction
HFrEF	heart failure with reduced ejection fraction
iNOS	inducible nitric oxide synthase
IRE1𝛼	inositol-requiring enzyme 1𝛼
L-NAME	N^ω^-nitro-L-arginine
MEF2	myocyte enhancer factor 2
MRA	mineralocorticoid receptor antagonist
NADPH	nicotinamide adenine dinucleotide phosphate
NFAT	nuclear factor of activated T cells
NFκB	nuclear factor kappa B
nNOS	neuronal nitric oxide synthase
NOS	nitric oxide synthase
NOX	NADPH oxidase
PDGF	platelet-derived growth factor
PKG	protein kinase G
RAAS	renin-angiotensin-aldosterone system
Rac1	Ras-related C3 botulinum toxin substrate 1
ROS	reactive oxygen species
SAUNA	salty drinking water/unilateral nephrectomy/aldosterone
sGC	soluble guanylate cyclase
SGLT2	sodium-glucose cotransporter 2
TGF-β	transforming growth factor-beta
Treg	regulatory T cell
VSMC	vascular smooth muscle cell
Xbp1(s)	(spliced) X-box-binding protein 1.
